# Who and how to screen for endogenous hypercortisolism in type 2 diabetes mellitus or obesity

**DOI:** 10.1007/s40618-024-02455-7

**Published:** 2024-10-01

**Authors:** Valentina Guarnotta, Carla Giordano, Giuseppe Reimondo

**Affiliations:** 1 Section of Endocrinology, Department of Health Promotion, Mother and Child Care, Internal Medicine and Medical Specialties “G. D’Alessandro” (PROMISE), University of Palermo, Piazza delle Cliniche 2, Palermo, 90127 Italy; 2 Internal Medicine, Department of Clinical and Biological Sciences, San Luigi Gonzaga Hospital, University of Turin, Orbassano, Italy

**Keywords:** Cushing’s syndrome, Overweight, Hyperglycaemia, Dexamethasone suppression test, MACS

## Abstract

**Purpose:**

The current review aims to summarize and discuss the prevalence of confirmed hypercortisolism in patients with diabetes mellitus or obesity, analysing the screening tests used and their accuracy, in order to better identify whether patients with diabetes mellitus and obesity should be screened for Cushing’s syndrome (CS) and how.

**Methods:**

A narrative review was performed including publications focusing on the current knowledge on prevalence of confirmed hypercortisolism in patients with type 2 diabetes mellitus (T2DM) or obesity and on screening tests used to detect CS.

**Results:**

The studies reviewed suggest that the prevalence of CS in patients with T2DM is variable, ranging from 0.6 to 9.3%. The most used screening test is the overnight cortisol after 1 mg of dexamethasone suppression test (DST), with a false positive rate ranging from 3.7 to 21%. The prevalence of CS among obese patients is generally about 1%, except for two studies which reported higher prevalence. For obese patients, 1 mg DST and late-night salivary cortisol are the most accurate screening tests for CS.

**Conclusions:**

Clinical expertise remains the mainstay to identify which subjects should be screened for CS. The evaluation of the clinical stigmata of CS and the combination with clinical comorbidities typical of CS are the stronger predictors of CS. In addition, we could hypothesize that in patients with T2DM, overnight 1 mg DST is the more accurate screening test for CS. By contrast, in patients with obesity both LNSC and overnight 1 mg DST could be equally used for the screening of hypercortisolism.

## Introduction

Cushing’s syndrome (CS) is a quite rare disease with an annual incidence of 2–3 per million [1, 2]. It is characterized by an endogenous cortisol hypersecretion that can be caused by an autonomous adrenal cortisol hypersecretion, adrenal adenoma or carcinoma, or by a pituitary or ectopic ACTH hypersecretion.

The diagnosis of CS can be delayed also many years after the onset of the first symptoms, due to the complexity of the syndrome that is characterized by many cardiovascular and metabolic disorders, but also by the need of combining more diagnostic test to confirm the syndrome, due to the variable accuracy of each test [3].

Recently, a difference between two entities mild autonomous cortisol secretion (MACS) and overt CS has been clarified.

MACS is defined as post-dexamethasone serum cortisol concentration above 50 nmol/L (> 1.8 µg/dL), without clinical signs of CS such as striae rubrae, bruising, lunaris face, muscle weakness, due to an adrenal tumour and therefore ACTH-independent [4–6]. By contrast, overt CS is associated with several comorbidities, including arterial hypertension, diabetes mellitus and increased fracture risk.

Comorbidities, such as diabetes mellitus, obesity, arterial hypertension, dyslipidaemia are very frequent in patients with overt CS, but also in the general population and therefore poorly discriminating [3, 7]. Conversely, weight gain, skin alterations and myopathy are the most frequent presenting symptoms of CS and can be considered more discriminating [8]. However, in some patients these signs and symptoms may be missing making more difficult the diagnosis. For these reasons, the selection of patients to be addressed for CS is relevant and depends on the experience of the examiner.

Currently, based on the guidelines of 2008, the screening for CS is recommended in those patients who show unusual symptoms for their age including osteoporosis and arterial hypertension, in patients who show high discriminatory signs and symptoms including easy bruising, facial plethora, proximal myopathy, purple striae with width > 1 cm, in children with weight gain combined with low height percentile or in patients with adrenal tumour [9].

However, in order to early identify patients with CS, many studies investigated the prevalence of CS in specific populations like subjects with obesity or T2DM, with contrasting results [10, 11].

The current review aims to evaluate the prevalence of confirmed CS in patients with diabetes mellitus and obesity, analysing the screening tests used and their accuracy, in order to better identify whether patients with diabetes mellitus and obesity should be screened for CS and how.

An extensive MEDLINE search was performed in July 2023 for the research question by the authors (VG and GR) independently, and discrepancies were resolved by discussion. A literature search was performed from 1990 to July 2023. The following search words were included: “Cushing’s Syndrome, diabetes, obesity, hypercortisolism”. Search terms were linked to the Medical Subject Headings (MeSH) when possible. Keywords and free words were used simultaneously. Additional articles were identified with manual searches and included thorough review of other meta-analyses, review articles, and relevant references.

## Prevalence of Cushing’s syndrome in patients with type 2 diabetes mellitus

Diabetes mellitus and glucose tolerance defects, including impaired fasting glycaemia (IFG) and impaired glucose tolerance (IGT), are very common in patients with CS and their prevalence is considered to range from 50 to 70% [12, 13].

Several studies evaluated the prevalence of CS, as MACS or overt hypercortisolism, in patients with diabetes mellitus, reporting discordant results (Table 1).

**Table 1:** Characteristics of screening studies of Cushing’s syndrome in patients with diabetes mellitus

Study First author	Prevalence	MACS or overt CS	Study design	Inclusion criteria	Exclusion criteria	Mean age (years)	Gender M/F	Screening test	Cut off	False positive rate	Other confirmatory tests
Leibowitz [11]	3.3%	Overt	Cross-sectional prospective study	BMI > 25 kg/m^2^; HbA1c > 9%		53.5 ± 1.6	26/64	1 mg DST	> 5 mcg/dL	2.2%	72 h DST; UFC; ACTH
Contreras [12]	2%	Overt	NA	NA	NA	NA	NA	UFC; 1 mg DST; Evening urinary cortisol	44 ng/mg creatinine	31%	NA
Catargi [13]	2%	Overt	Prospective screening study	BMI > 25 kg/m^2^; HbA1c > 8%	Cushingoid appearance, exogenous glucocorticoid, nephropathy	58.6	49/151	1 mg DST	> 2.1 mcg/dL	16.8%	LNSC; UFC; 4 mg DST
Chiodini [14]	9.3%	MACS	Prospective Case-control study	Hospitalized T2DM > 30 years; BMI > 19 and < 50 kg/m^2^ ; no insulin therapy in the first 2 years of disease	History of ketoacidosis, signs or symptoms of hypercortisolism and/or hyperandrogenism, chronic renal failure, acute illnesses, unawareness hypoglycaemia, alteration of sleep–wake cycle, depression, alcoholism, past or present glucocorticoid therapy or intake of drugs known to interfere with the pituitary-adrenal axis	60	124/165	1 mg DST	> 1.8 mcg/dL	6.1%	UFC; LNSC
Liu [24]	0	NA	Prospective	T2DM	Current or recent use of exogenous glucocorticoids; dementia; acute illness; use of medications known to interfere with cortisol testing, including carbamezepine; active night-shift employment; prior history of CS; autoimmune-mediated diabetes mellitus	61.8	141/0	LNSC	≥ 4.3 nmol/l	12%	UFC; LNSC; ACTH
Reimondo [15]	1%	Overt	Prospective	Newly diagnosed T1DM or T2DM	Signs and symptoms of hypercortisolism, drugs known to affect the HPA axis, alcohol abuse or current or previous history of major mood disorders	61	67/37	1 mg DST	> 4 mcg/dL	4%	2-day 2-mg DST
Caetano [25]	0	NA	Retrospective	T2DM	Age < 20 years; BMI < 25 kg/m2 Pregnancy and breast-feeding, Diagnosis of Cushing’s syndrome, depression, chronic renal, and/or hepatic insufficiency, and alcoholism, and use of drugs interfering with the diagnostic tests or laboratory assessment of cortisol	55.8 ± 8	34/69	1 mg DST; LNSC	> 1.8 mcg/dL > 253 ng/dL	0.9%	2-day 2-mg DST
Newsome [16]	0.6%	MACS	Prospective	T2DM > 1 year; Age > 18 years; BMI > 25 Kg/m^2^	Systemic or inhaled steroids, oral contraceptives, antidepressants or antipsychotics in the previous 3 months; cancer; suspected abuse of alcohol, mental incapacity, unwillingness or a language barrier; pregnancy	60.9	102/69	1 mg DST	> 1.8 mcg/dL	17.5%	UFC
Taniguchi [17]	2.6%	Overt	Prospective	Hospitalized T2DM patients	Cushingoid signs or symptoms; T1DM; diabetes secondary to other causes; alcoholism; depression; renal failure; acute illness	61	47/30	Midnight cortisol	> 5 mcg/dL	32.4%	0.5 mg DST; ACTH
Mullan [26]	0	NA	Prospective Case-control study	HbA1c > 7%; BMI > 25 Kg/m^2^ History of hypertension or BP > 140/90 mmHg	Exogenous steroids intake	58.9	119/91	LNSC	> 10 nmol/L	17.4%	2-day 2-mg DST
Gagliardi [27]	0	NA	Cross-sectional study	Age 40–75 years; BMI > 25Kg/m^2^CV > 80 cm Female; CV > 94 cm Male; HbA1c > 8%	Type 1, late-onset autoimmune or secondary diabetes mellitus; history of pituitary or adrenal disease; heart failure, moderate renal impairment, chronic liver disease, malignancy, alcohol dependence disorder or major psychiatric disorder, glucocorticoid therapy, acute illness, and shift-work.	58.5	60/40	LNSC	> 13 nmol/L	3%	1 mg DST; UFC
Murakami [18]	8.9%	MACS	Prospective	T2DM hospitalized patients	Age < 20 years; BMI < 16 kg/m^2^ o > 45 kg/m^2^; Cushingoid features; severe liver disease, serum creatinine > 1.3 mg/dL, infectious disease, malignant disease, steroid therapy	55.3	53/37	Midnight plasma cortisol	≥ 2.5 mcg/dL	54%	0.5 mg DST DDVAP test
Terzolo [19]	0.7%	Overt	Prospective	Age 18–70 years; BMI > 25Kg/m^2^; T2DM > 1 year	Cushingoid features, any severe acute illness, treatment with drugs known to affect the hypothalamus-pituitary-adrenal (HPA) axis or dexamethasone metabolism, current or previous history of alcohol, abuse or major mood disorders that required psychiatric intervention, history of recent surgery or trauma, and pregnancy	58.9 ± 8.9	428/385	1 mg DST	> 5 mcg/dL	4.1%	2-day 2-mg DST
Budyal [28]	0	NA	Prospective single centre study	T2DM outpatients	Cushingoid features, history of exogenous corticosteroid intake, pregnancy, women taking oral contraceptives within the last 6 weeks, anti-epileptic or antitubercular drugs, creatinine clearance > 60 ml/min or hepatic failure, depression, and alcoholism	55.1 ± 10.5	470/523	1 mg DST	> 1.8 mcg/dL	3.7%	2-day 2-mg DST
Gungunes [20]	0.7%	Overt	Prospective	HbA1c > 7% at least 3 months of long-acting insulin	Cushingoid features; any medications that affect hypothalamic–pituitary–adrenal axis or metabolism of dexamethasone; any serious acute diseases or kidney or liver failure; pregnancy and history of recent surgery or trauma	55.2 ± 9.2	57/220	1 mg DST	> 1.8 mcg/dL	4.6%	2-day 2-mg DST
Costa [21]	8.6%	MACS	Prospective	Age 18–80 years; Presence of microvascular or macrovascular complications, or with at least two modifiable cardiovascular risk factors	BMI > 40 Kg/m^2^; serum creatinine ≥ 180 mmol/L, presence of any serious concomitant disease limiting life expectancy; use of corticosteroids in the previous year; use of oral contraceptives or anti-epileptic medications within the last 6 weeks; depression; excessive alcohol intake; cushingoid features	58.4	141/252	1 mg DST	≥ 1.8 mcg/dL	21%	LNSC
Cansu [22]	2%	Overt	Prospective	Age > 40 years; T2DM for at least 2 years; BMI ≥ 25 kg/m^2^ Use of oral antidiabetic drugs or insulin	Use of exogenous glucocorticoids within the past 3 months; taking drugs that have an effect on cortisol levels or metabolism; depression, chronic renal failure, hepatic failure or dementia; alcoholics, pregnant or breastfeeding patients; acute diseases or malignant diseases; HbA1c > 8% without treatment; overt CS symptoms; bad sleep-wake cycle	56 ± 7	190/210	1 mg DST	> 1.8 mcg/dL	10.8%	2-day 2-mg DST Midnight serum cortisol
Steffensen [23]	5%	MACS	Cross-sectional study	T2DM	use of any type of glucocorticoid medication, psychiatric disease, alcohol intake > 14 unit/week for men and 7 units for women, and evidence of any acute medical condition	60 ± 10	232/152	1 mg DST	> 1.8 mcg/dL	15.6%	LDDST UFC

Abbreviations: DST dexamethasone suppression test, UFC urinary free cortisol, LNSC late night salivary cortisol, T2DM type 2 diabetes mellitus, DDVAP desmopressin test, NA not applicable, MACS mild autonomous cortisol secretion

### Studies which assessed the prevalence of overt CS

Leibowitz et al. reported for the first time 3 cases of confirmed CS (prevalence 3.3%) in 90 overweight and obese (BMI > 25 kg/m^2^) patients with poorly controlled diabetes mellitus (HbA1c > 9%) [14]. Four patients were initially identified by non-adequately suppressed cortisol values after overnight 1 mg dexamethasone suppression test (DST), with a false positive rate of 2.2%. Patients were further tested by 2 and 8 mg DST which confirmed 3 cases of CS. Two patients had a pituitary origin of CS and undergone pituitary surgery with improvement of clinical and biochemical parameters and only one had an adrenal CS, who undergone unilateral adrenalectomy.

Contreras et al. screened 48 overweight and diabetic patients vs. 40 normoglycaemic obese and 36 healthy subjects for hypercortisolism. They were screened by 24 h urinary free cortisol (UFC) and evening urinary cortisol together to overnight 1 mg DST. Authors reported a false positive rate of 1 mg DST in 31% of diabetic patients and 22% of obese subjects [15]. In addition, the diagnosis of confirmed pituitary CS was defined in only one diabetic patient, who performed pituitary surgery (prevalence 2%).

Catargi et al. conducted a prospective study on 200 patients with poorly controlled diabetes mellitus (HbA1c > 8%) reporting a prevalence of confirmed CS of 2% (4 out of 200 patients) [16]. Subjects were screened by overnight 1 DST (cut off 60 nmol/L or 2.18 mcg/dl) as first line screening test, with a false positive rate of 16.8%. As second step evaluation, cortisol and ACTH, UFC and 2 mg for 2 days of DST were performed together with radiological imaging. Three out of 4 patients had a pituitary adenoma, 2 undergone pituitary surgery with histological confirmation of CS and 1 who refused pituitary surgery undergone bilateral inferior sinus petrosal sampling for ACTH measurement. The fourth patient had an adrenal tumour, which was surgically removed with improvement of clinical and biochemical parameters.

Reimondo et al. screened 99 patients for CS with newly diagnosed diabetes mellitus by overnight 1 mg DST (cut off 110 nmol/l or 4 mcg/dL). After, patients were evaluated by 2 mg-2days DST and in patients with high suspicion for CS, ACTH, UFC, CRH test, midnight cortisol and radiological imaging were performed. Only 1 case out of 99 (prevalence of 1%) was a confirmed surgically proven pituitary CS. The reported false positive rate of 1 mg DST was of 4% [17].

Taniguchi et al. evaluated 77 hospitalized patients with diabetes mellitus first by midnight cortisol level (cut off 138 nmol/L or 5 mcg/dL) and after by overnight 0.5 mg DST and ACTH levels. Authors found a prevalence of 2.6% (2 out of 77 cases) of confirmed, surgically proven, pituitary CS [18].

Terzolo et al. evaluated the frequency of CS in a multicentric prospective study conducted on 813 Italian outpatients with T2DM screened by overnight 1 mg DST. Patients with a value more than 138 nmol/L or 5 mcg/dL underwent 2 mg-2days DST 3–6 months after first evaluation. In patients with not suppressed cortisol values, UFC, ACTH and pituitary MRI or abdomen TAC were performed to confirm diagnosis. Authors reported that 6 out of 813 patients (prevalence 0.7%) had a confirmed diagnosis of CS (5 adrenal and 1 pituitary dependent ones) [19]. The false positive rate of overnight 1 mg DST was 4.1%.

Gungunes et al. screened 277 outpatients with poor controlled T2DM (HbA1c > 7%) despite insulin therapy, by overnight 1 mg DST (cut off 50 nmol/l or 1.8 mcg/dL), with a false positive rate of 4.6%. Patients with not suppressed cortisol values underwent a 2 mg-2days DST and in 2 patients (prevalence 0.7%) CS was confirmed, 1 adrenal and 1 pituitary source, respectively [20].

Cansu et al. evaluated 400 diabetic patients divided according to HbA1c levels in group A (HbA1c ≥ 8%) and group B (≤ 6.5%). Patients were screened by overnight 1 mg DST (cut off ≥ 50 nmol/l or 1.8 mcg/dL) to detect hypercortisolism. In patients with not adequately suppressed cortisol values, a confirmatory 2 mg-2days DST (cut off ≥ 1.8 mcg/dL) and midnight serum cortisol (> 7.5 mcg/dL) were performed. ACTH, pituitary MRI, abdomen CT and 8 mg DST were further performed. Authors reported a prevalence of confirmed CS of 2% in group A (patients with poor controlled diabetes) [21], 4 patients with adrenal CS and 1 with pituitary CS.

Steffensen et al. screened 384 newly diagnosed T2DM patients by 1 mg overnight DST to identify overt CS. Patients with cortisol values after DST higher than 50 nmol/L, underwent a 2 mg-2days DST and UFC. Among all patients included in the study, authors found a 5% prevalence of MACS (20 out of 384) [22]. Notably, among 20 patients with hypercortisolism only 3 cases had a confirmed diagnosis of CS (1 with pituitary form and 2 with adrenal CS).

### Studies which assessed the prevalence of MACS

Chiodini et al. evaluated 294 hospitalized patients with type 2 diabetes mellitus (T2DM) over 30 years compared to a control group of non-diabetic subjects matched for age and BMI to identify the prevalence of MACS. The authors showed a prevalence of 9.4% of MACS in diabetic patients vs. 2% in the control group. Diagnostic criteria included plasma cortisol after overnight 1 mg DST more than 1.8 mcg/dl (50 nmol/l), with at least two of the following other tests including UFC higher than 60.0 mcg/24 h (165.6 nmol/24 h), plasma ACTH less than 10.0 pg/ml (2.2 pmol/l), midnight plasma cortisol more than 7.5 mcg/dl (207 nmol/l) and serum cortisol after corticotrophin-releasing hormone (CRH) stimulus during dexamethasone administration test more than 1.4 mcg/dl (38.6 nmol/l) [23]. All patients had radiological imaging. Authors reported a false positive rate of overnight 1 mg DST of 6.1%. Among the 30 patients with MACS identified, 21 were defined as adrenal subclinical hypercortisolism, 4 as pituitary subclinical hypercortisolism, 2 as ectopic subclinical hypercortisolism and 3 as undefined subclinical hypercortisolism. However, only 3 patients had surgical treatment and were histologically confirmed CS.

Newsome et al. screened 171 patients with T2DM, overweight, aged more than 18 years by overnight 1 mg DST (cut off 50 nmol/l or 1.8 mcg/dL). Thirty-one patients with not suppressed cortisol after 1 mg DST (false positive rate of 17.5%) were further evaluated by UFC (cut off 150 nmol/L), resulting in 3 patients with high UFC levels. In the end, only one patient resulted having a cyclical hypercortisolism, [24].

Murakami et al. screened 90 hospitalized diabetic patients for MACS. Diagnostic criteria were the presence of two or more following tests: midnight cortisol higher than 2.5 mcg/dL, cortisol value higher than 80 nmol/L or 3 mcg/dL after overnight 0.5 mg DST and positive desmopressin test. In patients with positive screening tests, CRH test, 8 mg of DST and pituitary MRI were performed, confirming 8 cases of MACS (prevalence of 8.9%) [25]. However, only 6 patients had radiological imaging with only 1 case with pituitary microadenoma, 2 cases with empty sella and 3 normal pituitary MRI. Unfortunately, no patients undergone pituitary surgery.

Costa et al. reported a prevalence of 8.6% of MACS in a population of 393 diabetic outpatients who underwent overnight 1 mg DST (cut off ≥ 50 nmol/l or 1.8 mcg/dL). In those patients with not suppressed cortisol values, LNSC in two samples (cut off > 0.35 mcg/dL) were additionally performed [26]. However, in this study the aetiology of hypercortisolism was not investigated.

### Studies which did not show increased prevalence of hypercortisolism

By contrast, other studies did not show increased prevalence of CS in patients with T2DM. Liu et al. evaluated 154 elderly men with T2DM compared with a control group of 54 men, by LNSC, with a reported false positive rate of 12%. The old age at enrolment in the study was maybe the main explanation of the missing CS diagnosis. In patients with high LNSC values, UFC and overnight 1 mg DST (serum cortisol > 50 nmol/l or 1.8 mcg/dL) were additionally performed. No cases of CS were found among the patients included in the study [27].

Caetano et al. screened 103 overweight outpatients with diabetes mellitus by LNSC and overnight 1 mg DST. Patients with upper quintile values for each test were further studied by 2 mg-2 days DST, without finding any cases of CS [28].

Similarly, Mullan et al. did not identify patients with CS among 201 overweight diabetic patients with HbA1c > 7% and arterial hypertension. LNSC was used as first screening test, with a false positive rate of 17.4%, followed by overnight 1 mg DST (cut off 60 nmol/L or 2.18 mcg/dl) [29].

Gagliardi et al. screened 106 overweight diabetic patients by LNSC for MACS. Three patients with high LNSC were further tested by overnight 1 mg DST (serum cortisol > 50 nmol/l or 1.8 mcg/dL) and UFC. No cases of CS were definitely found among patients included in the study [30]. A false positive rate of 3% for LNSC was reported.

A large single centre prospective study screened 993 T2DM outpatients by overnight 1 mg DST (cut off ≥ 50 nmol/l or 1.8 mcg/dL) to identify MACS. Thirty-three patients with positive DST underwent a 2 mg-2days DST without finding any cases of CS [31].

## Prevalence of Cushing’s syndrome in patients with obesity

Central obesity is very common in patients with CS and it is characterized by a redistribution of adipose tissue from limbs to abdominal region, face, neck and trunk. For this reason, it may be hypothesized that patients with central obesity should be screened for CS.

Several studies have evaluated the reliability of CS screening in patients with obesity, with discordant results (Table 2).

**Table 2:** Characteristics of screening studies of Cushing’s syndrome in patients with obesity

Study	Prevalence	Study design	Inclusion criteria	Exclusion criteria	Mean age (years)	Gender M/F	Screening test	Cut off	Diagnostic test accuracy	Other confirmatory tests
Ness-Abramof [33]	3.5%	Prospective	BMI > 30 kg/m^2^	NA	42.9 ± 13.2	13/73	1 mg DST	> 3 mcg/dL	False positive 2.3%	UFC; 2-day 2-mg DST
Pasquali [34]	0	Prospective	Age 18–65 years	NA	34.7 ± 11.0 (women) 39.7 ± 13.6 (men)	36/51	1 mg DST	> 5 mcg/dL	NA	NA
Baid [35]	0	Cross-sectional prospective	Overweight or obese subjects with at least two additional features of Cushing’s syndrome	Weight > 159 kg; serum creatinine > 2.6 mg/dl; pregnancy; serious medical conditions that might alter pituitary-adrenal function; and recent or anticipated use of oral or injected glucocorticoids, black licorice, chewing tobacco, phenytoin, barbiturates, loperamide, or opiates	48 ± 12 (women) 50 ± 12 (men)	100/269	UFC; LNSC; 1 mg DST	> 45 mcg/24 h > 170 ng/dL (RIA) > 100 ng/dL (LC-MS) ≥ 1.8 mcg/dL	Specificity 96% Specificity 84% Specificity 95% Specificity 90%	NA
Tiryakioglu [36]	8.7%	Retrospective	BMI > 25 kg/m^2^	Exogenous glucocorticoid intake, alcoholism, obvious depression obvious depression, or pregnancy; creatinine clearance < 30 ml/min; acute illness or sleep disorders; diabetes mellitus	44.4 ± 13.3	21/129	UFC	> 100 mcg/24 h	False positive rate 15.3%	1 mg DST
Jankovic [37]	< 0.6%	Retrospective	Obese subjects pre-bariatric surgery	NA	41 ± 12	104/329	1 mg DST	> 3 mcg/dL	NA	NA
Sahin [38]	0.5%	Prospective	BMI > 30 kg/m^2^	Cushingoid features; exogenous glucocorticoid intake, serious medical conditions that might alter pituitary-adrenal function, antiepileptics, estrogens, alcohol dependence, depression, psychiatric conditions, pregnancy, creatinine clearance < 30 mL/min	37.8 ± 13.4	43/311	ACTH; Cortisol; 1 mg DST	NA	False positive rate 1 mg DST 1.4%	2-day 2-mg DST
Fierabracci [39]	0.8%	Prospective	Age 18–65 years scheduled for bariatric surgery	NA	44 ± 12	174/609	1 mg DST	> 3 mcg/dL	NA	2-day 2-mg DST
Alhambra Exposito [40]	0.25%	Retrospective	Age 18–60 years; BMI ≥ 35 kg/m^2^ Scheduled for bariatric surgery	Cushingoid features; depression or psychiatric conditions; drugs that may affect pituitary-adrenal function	41.9 ± 10.5	91/308	1 mg DST	≥ 1.8 mcg/dL	False positive rate 1 mg DST 5%	UFC
Javorsky [44]		Retrospective	CS neo diagnosis or recurrence after bariatric surgery	NA	36	0/16	NA	NA	NA	NA
Yavuz [41]	0.77%	Retrospective	BMI > 40 kg/m^2^ scheduled for bariatric surgery	Alcoholism; exogenous glucocorticoid intake; pregnant women	42 ± 10	238/799	1 mg DST	≥ 1.8 mcg/dL	Specificity 96.8%	2-day 2-mg DST
Atar [42]	1.4%	Retrospective	BMI ≥ 30 kg/m2	Previous diagnosis of CD, CS or ACS, patients < 18 years of age, pregnant women, patients with liver and kidney failure (GFR < 60 ml/min), patients using oral, parenteral or topical glucocorticoids, oral contraceptives or drugs that could induce CYP 3A4 enzymes, patients with type-1 DM, and those with a history of psychiatric illnesses, including major depression and obsessive-compulsive disorder	46.4 ± 14.2	130/683	1 mg DST	> 1.8 mcg/dL	False positive rate 0	2-day 2-mg DST
Baldane [43]	0.75%	Retrospective	BMI ≥ 40 kg/m2 Pre-bariatric surgery	Pregnant women, patients with alcohol abuse, exogenous glucocorticoid use, kidney or liver diseases or patients previously suspected with CS or adrenal mass	40 ± 12	208/545	1 mg DST	≥ 1.8 mcg/dL	False positive rate 2.4%	UFC; Midnight serum cortisol

Abbreviations: DST dexamethasone suppression test, UFC urinary free cortisol, LNSC late night salivary cortisol

### Studies which assessed the prevalence of overt CS

Ness-Abramof et al. screened 86 patients with obesity (combined with arterial hypertension or diabetes mellitus), by 1 mg overnight DST (cut off > 3 mcg/dL or 80 nmol/L), confirming CS in 5 out 86 patients (prevalence 3.5%). Among 5 patients with confirmed CS, 3 had a pituitary surgery, 1 had a macronodular adrenal hyperplasia and the other 1 had a cyclical CS [32].

Pasquali et al. evaluated the suppressibility of the hypothalamic-pituitary-adrenal axis in 34 normal-weight and 87 obese subjects by overnight 1 mg DST and three different weight-adjusted dexamethasone doses [33]. Authors did not find any differences in serum cortisol between the two groups after the standard 1-mg DST (all patients suppressed cortisol levels to < 138 nM or 5 mcg/dL) and the adjustment of dexamethasone dose to body weight did not change the sensitivity of the test, even in obese patients.

Tiryakioglu et al. screened 150 obese people by UFC (cut off 100 mcg/24 h) and overnight 1 mg DST (cortisol cut off ≥ 1.8 mcg/dL or 50 nmol/L) confirming, by histological exam, CS in 14 out of 150 patients (prevalence 8.7%) [34].

Fierabracci et al. screened 783 obese patients before bariatric surgery by overnight 1 mg DST (cortisol cut off > 3 mcg/dL or 80 nmol/L) [35]. Patients with abnormal results underwent a 2 mg-2days DST finding a prevalence of 0.8% of confirmed CS.

Sahin et al. screened 354 obese patients by overnight 1 mg DST (cortisol cut off ≥ 1.8 mcg/dL or 50 nmol/L) reporting a false positive rate of 1.4% [36]. Patients who failed to suppress cortisol values after DST, performed a 2 mg-2days DST, with a prevalence of 0.5% of confirmed CS.

Alambra Exposito et al. retrospectively evaluated 399 patients with obesity who were screened for CS before undergo bariatric surgery, by overnight 1 mg DST (cortisol cut off ≥ 1.8 mcg/dL or 50 nmol/L). Patients who failed to suppress cortisol after DST underwent UFC. Among the 399 patients, only one case of CS was confirmed (prevalence 0.25%) [37].

A low prevalence (0.77%) of confirmed CS was also reported by Yavuz et al. who screened 1037 class 3 obese patients before bariatric surgery by overnight 1 mg DST (cortisol cut off ≥ 1.8 mcg/dL or 50 nmol/L) [38]. Patients with not adequately suppressed cortisol values were further evaluated by two samples of UFC (cut off > 100 µg/day), 2 mg-2days DST, ACTH and midnight serum cortisol values.

Atar et al. screened 813 obese patients finding hypercortisolism in 39 patients with a prevalence of 5.4%. Patients were screened by overnight 1 mg DST (cortisol cut off ≥ 1.8 mcg/dL or 50 nmol/L) [39] and after confirmed by ACTH, 2 mg-2days DST, UFC and LNSC. However, only 4 patients with pituitary disease and 3 with adrenal tumours had a surgically confirmed diagnosis of CS with a prevalence of 0.8%.

Similarly, Baldane et al. screened 753 class 3 obese patients, before bariatric surgery, by overnight 1 mg DST (cortisol cut off ≥ 1.8 mcg/Dl or 50 nmol/L) showing inadequate suppression in 3.18% of patients, reporting a specificity of 97.5%. CS was further confirmed by additional tests in 0.75% of patients [40].

In the end, Javorsky et al. retrospectively reviewed 16 patients with CS who were diagnosed after performance of bariatric surgery, suggesting that CS may be unrecognized in patients undergoing bariatric surgery [41].

### Studies which did not show increased prevalence of hypercortisolism

Baid et al. conducted a large prospective study on 369 overweight and obese patients who were screened by LNSC, UFC and overnight 1 mg DST to identify CS. Patients with abnormal results underwent 2 mg-2days DST and dexamethasone-CRH test without confirming any cases of CS [42].

Jankovic et al. screened 433 obese patients before undergoing bariatric surgery by overnight 1 mg DST (cortisol cut off > 3 mcg/dL or 80 nmol/L) and further by UFC and 2 mg-2days DST without confirming any cases of CS [43].

## Discussion

We reviewed the current knowledge of the prevalence of confirmed CS in patients with diabetes mellitus or obesity.

We evaluated 18 and 12 original studies which screened patients with T2DM or obesity, respectively, for CS.

With regard to diabetes mellitus, in 5 out of 11 studies, authors did not find any cases of CS. In the remaining 6 studies the prevalence of CS ranged from 0.6 to 9.3%. Higher prevalence was reported by Chiodini et al. [23]. , Murakami et al. [25]. and Costa et al. [26]. who reported a prevalence of CS of 9.3, 8.9 and 8.6%, respectively. Chiodini et al. and Murakami et al. included hospitalized patients, while Costa et al. evaluated outpatients. Patients included in the study by Murakami et al. had not diabetic complications, patients included in the study by Chiodini et al. had a longstanding diabetes mellitus without need of insulin therapy in the first 2 years of disease and those included in the study by Costa et al. had diabetic microvascular complications. The higher prevalence of hypercortisolism in these studies could also depend on the diagnostic criteria used in both screening and confirmatory tests, in which sensitivity was privileged over specificity. Further, all these studies did not report the prevalence of confirmed CS, but only of MACS. Indeed, in the study conducted by Chiodini et al. only 3 patients had a surgical confirm of CS, while in the studies conducted by Murakami et al. and Costa et al. no surgically confirmed CS cases were reported. This aspect could explain why these 3 studies showed higher prevalence than the others, which showed a mean prevalence of 1–2% of confirmed CS.

Further, the higher rate of hypercortisolism in hospitalized patients may represent a bias due to the stress of hospitalization which could be a risk for false positive results.

In addition, if we would consider the total of patients included in the current revision, we could calculate a mean prevalence of 2% of confirmed CS (about 74 out 3336), similar to that reported by the studies which evaluated the prevalence of only confirmed CS.

In the metanalysis performed in 2016 by Steffensen et al., a prevalence of hypercortisolism and confirmed CS, in patients with T2DM, was reported in 3.4% and 1.4% respectively [44]. Our revision of the data is in accordance with metanalysis by Aresta et al. who showed that studies which used more specific and less sensitive screening criteria had the lowest prevalence (0.7–1.3%), while those using fewer specific criteria had a prevalence rate of about 10%. In addition, Aresta et al. concluded that patients with T2DM with arterial hypertension (notably those treated with at least 2 anti-hypertensive drugs), requiring insulin therapy and those with diabetic microvascular and macrovascular complications should be screened for hidden hypercortisolism [45].

Another aspect we focused on, is which test could be the more appropriate to screen patients with T2DM for CS. In 11 out of 18 studies reviewed, cortisol after 1 mg of DST was the most used. In these studies, different cut offs were used. In 7 out of 11 studies the used cut off was ≥ 1.8 mcg/dL with a false positive rate ranging from 3.7 to 21%. In the remaining 4 out of 11 studies, which used higher cut offs (3 or 5 mcg/dL), the false positive rate was lower, ranging from 2.2 to 16.8%. In 4 out 18 studies, LNSC was used as first line screening test, with a false positive rate ranging from 3 to 19.4%. Two out 18 studies screened CS by midnight serum cortisol, with higher false positive rate ranging from 32 to 54%. The other remaining study combined more screening tests, reducing the number of false positive results.

Interestingly, Steffensen et al. compared the accuracy of LNSC, 1 mg DST and UFC as screening tests for hypercortisolism, showing that LNSC had a lower specificity than 1 mg DST in patients with diabetes mellitus [22]. Another study by Steffensen et al. compared the accuracy of LNSC and overnight 1 mg DST in detecting CS in patients with T2DM [44]. Authors included 382 newly diagnosed patients with T2DM who performed LNSC (cut off > 3.6 nmol/L) and overnight 1 mg DST (cut off > 50 nmol/L or 1.8 mcg/dL). 86% of patients who performed LNSC had high cortisol values, while only 22% of them had not suppressed cortisol values after DST. LNSC showed sensitivity of 85% and specificity of 14%, positive predictive value of 22%, negative predictive value of 76% and overall accuracy of 30% compared to DST. Both LNSC and DST values were not associated with HbA1c, BMI and age. Steffensen et al. concluded that LNSC was characterized by very low specificity and poor positive predictive value as compared to the DST, resulting in an overall low accuracy.

With regard to the screening for CS in patients with obesity, we reviewed 12 studies. Among them, 2 studies did not find any cases of CS in obese patients. The other studies reported a low prevalence of about 1% or less, except for those conducted by Ness-Abramof et al. [32]. , which reported a prevalence of 3.5% and by Tiryakioglu et al. [34]. which reported a prevalence of 8.7%. However, Tiryakioglu et al. reported the prevalence of MACS. Indeed, the calculated prevalence of surgically confirmed CS was significantly lower, about 0.8%.

A recent systematic review and metanalysis aimed to evaluate the prevalence of several endocrine disorders in obesity showed that the pooled prevalence of hypercortisolism in obese subjects from a random effect was 0.9% [46]. The mean BMI was not correlated to the reported prevalence.

Belaya et al. evaluated the diagnostic performance of LNSC in detecting CS in 123 obese patients and 98 healthy subjects reporting that a cut-off value of 9.4 nmol/l can differentiate CS among obese and overweight patients with sensitivity of 84.4% (95% CI 71.2–92.2), specificity of 92.3% (95% CI 84.2–96.4), and diagnostic odds ratio of 65.1 (95% CI 20.4–207.6) [47]. More recently, another study evaluated the specificity of LNSC for the screening of CS on 157 patients, including 40 healthy subjects, 83 obese subjects and 34 patients with histopathologically proven CD, on three different cut-offs. Analysing healthy subjects and obese patients, against the CD group, ROC analysis showed a sensitivity of 67.6% and specificity of 85.4% for a cut-off value of 12.3 nmol/L [48]. Similarly, Ceccato et al. showed that LNSC had a high diagnostic accuracy to exclude hypercortisolism in patients with normal cortisol levels, also measured in chemiluminescence, even though mass spectrometry could reduce the number of false-positive results [49].

Lammert et al. evaluated the specificity of overnight 1 mg DST in 278 obese patients screened for CS, showing a low prevalence (0.8%) of hypercortisolism in their population, with a good specificity 92% of the test (< 50 nmol/L). After exclusion of drugs interfering with CYP3A4, the specificity increased to 94.9% [50]. Similar data were reported by Sahin et al. who reported 1.4% of false positive test of overnight 1 mg DST [36]. More recently, Yavuz et al. also evaluated the specificity of overnight 1 mg DST for the screening of obese patients, reporting a value of 96.8% (cut off 1.8 mcg/dL) [38]. Almost all studies used the cortisol after 1 mg DST as first line screening test, with low false positive rates, except for the study conducted by Tiryakioglu et al., which used UFC as first screening test, with a false positive rate of 15.3%. The studies which evaluated LNSC and overnight 1 mg DST accuracy showed that LNSC had a good specificity for the screening of CS, ranging from 85.4 to 92.3% [47, 48]. Similarly, overnight 1 mg DST showed higher specificity for the screening of CS in patients with obesity and lower false positive rates [50]. Further, Ellis et al. compared LNSC with UFC and overnight 1 mg DST in 40 obese T2DM patients [51]. Authors reported a specificity of 70% of LNSC, 90% of UFC and 72% of 1 mg DST, respectively. The specificity of LNSC was significantly less than UFC (*p* = 0.039), but similar to 1 mg DST.

We should also mention that the high false positive rate of hypercortisolism reported in many studies on obese patients could be explained by the effort to counteract local cortisol excess in obesity. Indeed, an inverse significant relationship has been reported between cortisol levels (UFC and 1 mg DST) and BMI values [52].

According to the above-mentioned studies, we could hypothesize that in patients with T2DM, overnight 1 mg DST is the more accurate screening test for CS. By contrast, in patients with obesity both LNSC and overnight 1 mg DST could be equally used for the screening of hypercortisolism. Therefore, in obese patients, LNSC could be a more accurate screening test for hypercortisolism, than in patients with T2DM.

In conclusion, the prevalence of CS both in patients with T2DM and in patients with obesity was very different among the studies included in the current review. Indeed, patients’ populations and study designs were much different, the typical phenotypic characteristics of cortisol excess were generally exclusion criteria, some studies reported the prevalence of MACS, while others of confirmed CS and the biochemical work-up was very heterogeneous using LNSC, UFC and cortisol after 1 mg DST as screening tests. In addition, among the studies which used cortisol after overnight 1 mg DST as first-line screening test, the cut-offs were very different ranging from 1.8 to 5 mcg/dL, making heterogeneous the results and requiring further confirmatory tests. Another limitation was also the setting of the centre, indeed high volume or academic centre might centralize peculiar resistant subjects, thus increasing the likelihood of rare disease discovery.

It is clinically plausible that patients with additional comorbidities over T2DM should potentially have an increased risk of hidden hypercortisolism. The evaluation of the clinical stigmata of CS and the combination with comorbidities typical of CS may be stronger predictors of unknown CS and are more important than the screening test alone, which is not cost-effective. Currently, the available data are not sufficiently satisfactory to suggest a wide and indiscriminate screening in patients with T2DM or obesity. Further, obesity alone can be a pitfall because it can be a condition of HPA axis activation and should be always discriminated the central obesity combined with thin arms and legs, highly suggestive for hypercortisolism, from the non-specific generalized obesity.

Therefore, in the era of artificial intelligence, the human intelligence remains the best cost-effective screening test in identifying which subjects should be screened for hypercortisolism.
