# Correction: Who and how to screen for endogenous hypercortisolism in type 2 diabetes mellitus or obesity

**DOI:** 10.1007/s40618-024-02487-z

**Published:** 2024-11-09

**Authors:** Valentina Guarnotta, Carla Giordano, Giuseppe Reimondo

**Affiliations:** 1 Section of Endocrinology, Department of Health Promotion, Mother and Child Care, Internal Medicine and Medical Specialties “G. D’Alessandro” (PROMISE), University of Palermo, Piazza delle Cliniche 2, Palermo, 90127 Italy; 2 Internal Medicine, Department of Clinical and Biological Sciences, San Luigi Gonzaga Hospital, University of Turin, Orbassano, Italy

**Correction: Journal of Endocrinological Investigation**

**https://doi.org/10.1007/s40618-024-02455-7**.

In the original version of this article, Some part of the Funding information was missing. The funding information was previously read "This study was funded by “Recordati Rare Diseases”. Open access funding provided by Università degli Studi di Palermo within the CRUI-CARE Agreement." should have been "This study was funded by “Recordati Rare Diseases”. Open access funding provided by Università degli Studi di Palermowithin the CRUI-CARE Agreement - Open Access funding for this article was provided by Recordati Rare Diseases Italy S.r.l. The funder had no role in the preparation, review, or approval of the manuscript.”

The original article has been corrected.

